# Homocysteine Level Related to Age Is Associated With Embryo Quality in Women Who Had IVF With Diminished Ovarian Reserve

**DOI:** 10.3389/frph.2022.886277

**Published:** 2022-07-11

**Authors:** Haiyan Wang, Aiqing Han, Shiyuan Jiang, Dan Cao, Yangyu Jiang, Lin Sun, Na Zou, Shiying Tao, Xiaoou Xue, Xiaoguang Shao, Jian Li

**Affiliations:** ^1^Department of Histology and Embryology, School of Traditional Chinese Medicine, Beijing University of Chinese Medicine, Beijing, China; ^2^Reproductive and Genetic Medical Center, Dalian Women and Children's Medical Group, Dalian, China; ^3^School of Management, Beijing University of Chinese Medicine, Beijing, China; ^4^Department of Pathology, Dalian Municipal Women and Children's Medical Center (Group), Dalian, China; ^5^Dongzhimen Hospital, Beijing University of Chinese Medicine, Beijing, China

**Keywords:** homocysteine, *in vitro* fertilization (IVF), diminished ovarian reserve (DOR), embryo quality, aged

## Background

Infertility has become a worldwide public health problem, affecting 12.5 to 15 in 100 couples of reproductive ages annually in China (1). Diagnosis of diminished ovarian reserve (DOR), a predominant contributor to infertility, has increased from 19% in 2004 to 26% in 2011 in the United States, while similar trends have emerged in China (2). Aging is directly associated with physiological DOR, whereas endometriosis and some diseases contribute to pathological DOR (3). However, the pathophysiological mechanisms of DOR remain unclear.

The assisted reproductive technique (ART) has been broadly used to improve reproductive outcomes for women with infertility. Nevertheless, DOR is also a big challenge in ART, which addresses reductions in oocyte quantity and quality, poor ovarian response (POR), high cancellation rate, and recurrent pregnancy loss (RPL) (4, 5).

The number of oocytes and high-quality embryos acquired was crucial across all *in vitro* fertilization (IVF) laboratories in ART. Progress is being made in exploring the pathophysiologic mechanisms of poor-quality embryos and discovering therapeutic strategies for improving the embryo quality of women with DOR (6, 7).

Recently, investigators have demonstrated some potential correlations between serum homocysteine (Hcy) level and embryo quality (8). In brief, Hcy concentration correlates with oocyte and embryo qualities in patients with infertility and polycystic ovary syndrome (PCOS). Hyper-homocysteinemia (hHcy) has been identified as a risk factor for unexplained infertility (9, 10). Furthermore, aging (≥39 years) and high levels of plasma Hcy are strongly associated with thrombotic events during IVF. Mild-to-moderate hHcy is an independent risk factor for thromboembolic disorders (11), implantation failure in patients undergoing IVF (9), and early pregnancy loss (12). However, to our knowledge, no study has yet assessed the correlation between serum Hcy level and embryo quality in patients with DOR undergoing IVF.

In the presented study, we analyzed the medical records of infertile women with DOR who underwent ART at our center to reveal the possible correlation between clinical characteristics and embryo quality. Thus, our objective was to provide evidence for predicting embryo quality and suggesting potential therapeutic approaches by evaluating Hcy levels in patients undergoing IVF treatment.

## Methods

### Data Source

**Figure 1:**
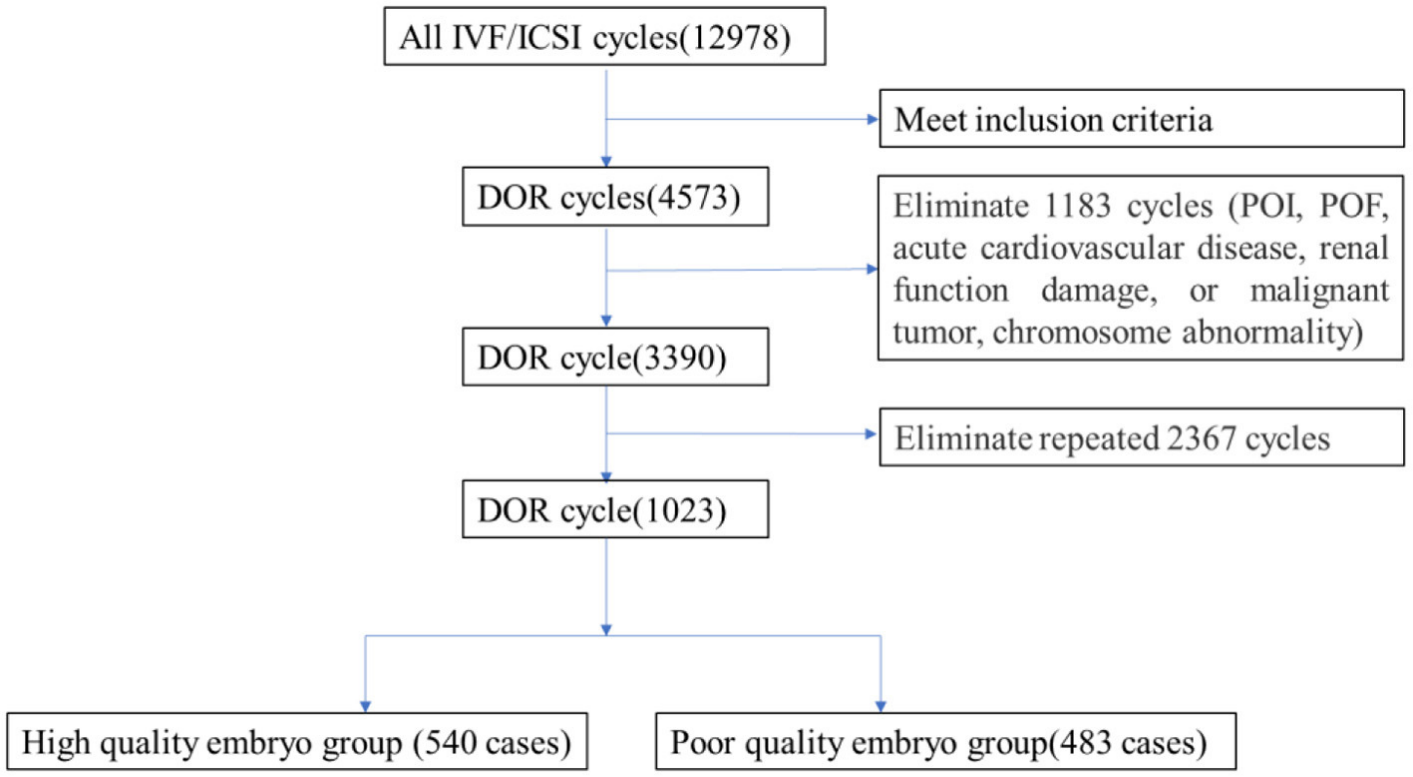
The flowchart.

A retrospective cohort study was conducted using 3,390 medical record data on IVF cycles from Dalian Municipal Women and Children's Medical Center (Group) from January 2015 to December 2020. The flowchart is shown in Figure 1. The inclusion criteria were (1) women aged 20~49 years old, (2) patients who underwent IVF/ intracytoplasmic sperm injection (ICSI) for the first time, and (3) patients with serum basal follicle-stimulating hormone (FSH) ≥ 10 mIU/ml, anti- Müllerian hormone (AMH) < 1.1 ng/ml, or antral follicle count (AFC) < 7 (1–3 months of the IVF cycle).

Patients were excluded if any of the following conditions were present: (1) premature ovarian failure (POF) or primary ovarian insufficiency (POI), (2) acute cardiovascular disease, renal function damage, or malignant tumor, and (3) chromosome abnormality by preimplantation genetic screening.

### Stimulation and IVF Procedure

Stimulation protocols were selected based on the individual patient's conditions. Standard ovarian stimulation with gonadotrophins was performed using gonadotrophin-releasing hormone (GnRH) antagonist protocol, mini-stimulation protocol, or natural protocol. Oocyte retrieval was performed 34~36 h after triggering with hCG, a GnRH agonist, or a combined hCG and GnRH agonist under transvaginal ultrasound guidance.

Cases with no oocyte obtained or ovulation occurring at the time of egg collection were excluded. Oocytes were fertilized using either IVF or intracytoplasmic sperm injection (ICSI) depending on the semen parameters of the husbands per the standard protocols in the centers. The fertilization was confirmed by the presence of two pronuclei at 16~18 h after conventional insemination or ICSI. Three days after oocyte retrieval, an embryo with at least seven cells and Grades 1 and 2 was defined as good quality. Embryos with at least six cells and fragments of <50% were frozen. All good embryos were frozen or vitrified using the Cryotop method as cleavage-stage embryos on Day 3, or as blastocysts on Day 5 or 6 according to the standard operating protocol.

### Embryo Quality Assessment

The DX1800 automatic chemiluminescence instrument (Beckman Coulter Company, USA) was used for hormone determination, and an automatic biochemical analyzer (Hitachi Company, Japan) was used to detect blood lipid levels. The selection of fertilization method and embryo score, according to pronuclear stage score, development speed, number of blastomeres, size, morphology, cytoplasm, fragment ratio, and embryo quality score of the cleavage stage, was divided into four grades: Grade 1 embryo: blastomere equality, regular morphology, bright, and no fragment; Grade 2 embryo: blastomere unequal in size and/or fragmentation of <10%. Grade 3 embryo: 10–50% fragments; and Grade 4 embryo: fragmentation >50%. Embryos with more than six cells in Grades 1-2 on Day 3 after egg collection were defined as high-quality embryos. Embryos with <30% fragments are available and can be transplanted or frozen (Figure 1).

### Data Collection and Handle

A total of 1,023 data met the criteria. The data were divided into poor-quality embryos group and high-quality embryos group according to the embryo status. We summarized the demographic and clinical characteristics (age, infertility years, infertility type, AFC, and BMI), medical history, reproductive hormone parameters (AMH, FSH, LH, PRL, and E_2_), and carbohydrate and lipid metabolic indexes (CHO, TG, LP, LDL, HDL, fasting insulin (FINS), Glucose, and Hcy).

### Statistical Analyses

Descriptive statistics were presented as mean ± standard deviation (SD) for continuous variables. Categorical variables were presented as frequency and percentage (%). Normally distributed data were analyzed using Student's *t*-test. Non-parametric variables were compared using the Mann-Whitney *U* test. Categorical variables were compared using the chi-square or Fisher's exact tests. Binary logistic regression analysis with backward stepwise elimination of candidate variables was used to measure the association between independent and outcome variables. Results were presented as Odds ratios (*OR*s) with 95% confidence intervals (*CI*s) and area under the curves (AUCs).

Data were analyzed using SPSS version 20 (IBM SPSS, Inc., Chicago, IL, USA). Comparisons were interpreted as significant when associated with *P* < 0.05. The ROC curves and AUC values were calculated using the pROC package of R statistical software (Version 4.1.0). The scatter box diagram was drawn by python software (Version 3.8.3).

## Results

### Patient Characteristics

A total of 1,023 patients with DOR were included in this study. Of these, 540 cases were with high-quality embryos (the number of high-quality embryos, number ≥1) and 483 cases were with poor-quality embryos (no high-quality embryos, number = 0). The demographic characteristics of the two groups were similar (Table 1). However, the level of serum Hcy was significantly higher in the poor-quality embryo group compared with the high-quality embryo group (*P* < 0.05). In addition, the levels of serum AMH and PRL were statistically different between the two groups.

**Table 1:** Characteristics of the patients with DOR.

**Characteristics**	**Number of high-quality embryos**		**z/χ^2^**	***P*-value**
	**Poor-quality (0) (*n* = 483)**	**High-quality (≥1) (*n* = 540)**		
Age, y	36.78 ± 4.79	36.19 ± 4.53	−1.68	0.093
Infertility years, y	3.71 ± 3.26	3.48 ± 2.81	−0.49	0.621
Infertility type				
Primary, n (%)	244 (50.5%)	258 (47.8%)	0.766	0.382
Secondary,n (%)	239 (49.5%)	282 (52.2%)		
AFC, number	3.44 ± 1.28	3.62 ± 1.46	−0.97	0.331
BMI, kg/m^2^	23.15 ± 3.54	22.98 ± 3.28	−0.50	0.618
AMH, ng/mL	0.78 ± 0.94	0.81 ± 0.49	−2.38	***0.017***
FSH, mIU/mL	9.73 ± 4.43	9.07 ± 3.62	−1.83	0.067
LH, mIU/mL	5.10 ± 2.51	4.97 ± 2.30	−0.35	0.728
PRL, ng/ml	19.35 ± 30.34	19.3 ± 26.45	−2.15	***0.031***
E_2_, pmol/ml	142.95 ± 72.59	147.68 ± 66.88	−1.13	0.258
CHO, mmol/L	4.74 ± 0.84	4.70 ± 0.87	−0.99	0.324
TG, mmol/L	1.36 ± 0.94	1.30 ± 0.84	−1.36	0.174
LP, g/L	192.26 ± 223.71	157.95 ± 214.27	−1.63	0.104
LDL, mmol/L	2.76 ± 0.67	2.76 ± 0.68	−0.09	0.931
HDL, mmol/L	1.49 ± 0.32	1.48 ± 0.31	−0.73	0.464
FINS, μIU/mL	9.77 ± 3.89	10.14 ± 3.89	−1.45	0.148
Glucose, mmol/L	5.14 ± 0.71	5.12 ± 0.65	−0.15	0.885
HCY, μmol/L	9.22 ± 3.70	8.57 ± 2.41	−2.31	***0.021***

*Data presented as mean ± SD or n (%)*.

*The t-test or the Mann-Whitney U tests were used in the continuous data and the chi-square test or Fisher's exact tests were used in the categorical data. P values of bold italic are less than 0.05*.

### Relationship Between Hcy and High-Quality Embryo Number

**Figure 2:**
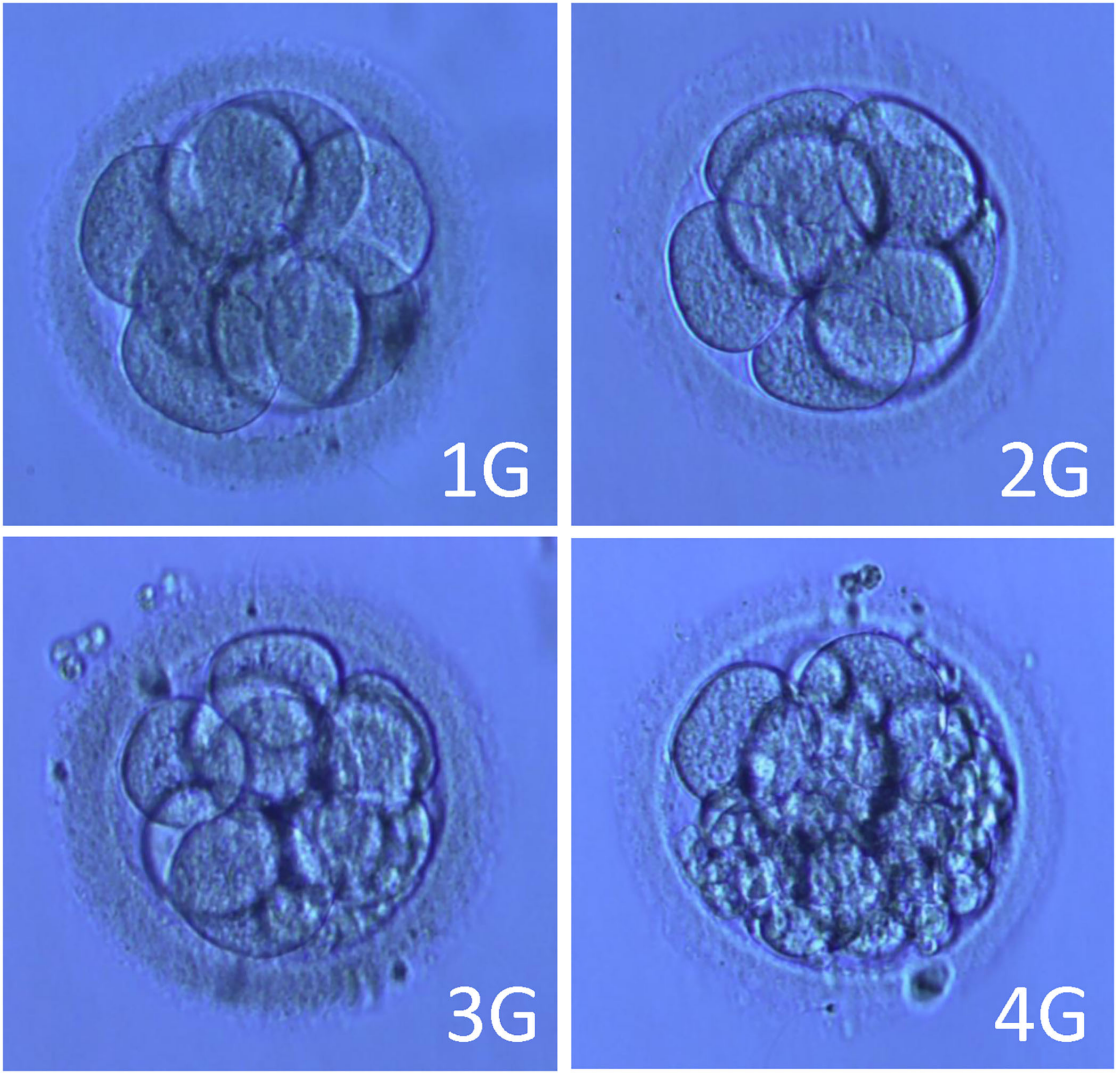
Pictures of embryos of different grades. Note: 1G (Grade 1 embryo): blastomere equality, regular morphology, bright, no fragment. 2G (Grade 2 embryo): blastomere unequal in size and/or fragmentation <10%. 3G (Grade 3 embryo): 10–50% fragments. 4G (Grade 4 embryo): fragmentation > 50%.

Impressed with the higher Hcy level in the poor-quality embryo group, we analyzed the relationship between the number of high-quality embryos and the level of serum Hcy. The level of serum Hcy negatively correlated with the number of high-quality embryos (Figure 2). A high level of serum Hcy might be one of the key factors that triggers the decline in embryo quality.

To further evaluate the Hcy levels in patients with different embryo quality, we perform subgroup analysis. Patients with serum Hcy**>**15 μmol/L were at higher risk of having poor-quality embryos (82.1%) compared to patients with serum Hcy <8 μmol/L (43.9%) and at 8~15 μmol/L (48.1%) (Table 2; χ^2^ = 15.859, *P* < 0.001).

**Table 2:** Comparison of high-quality embryo rate among different Hcy groups.

**Hcy Level**	**Embryos quality**		***χ^2^***	***p***
	**Poor-quality (0) (*n* = 460)**	**High-quality (≥1) (*n* = 515)**		
≤ 8 μmol/L, n (%)	193 (43.9%)	247 (56.1%)	15.859	<0.001
8~15 μmol/L, n (%)	244 (48.1%)	263 (51.9%)		
≥15 μmol/L, n (%)	23 (82.1%)	5 (17.9%)		

*P < 0.05 was considered to be statistically significant*.

### Logistic Regression Analyses

Since the above results showed a negative correlation between the serum Hcy and high-quality embryos, a logistic regression analysis was performed to measure the association between independent variables and high-quality embryos. Patients who were older (*OR* = 0.960, 95% *CI* = 0.926–0.996, *P* < 0.05) or with higher Hcy level (*OR* = 0.901, 95% *CI* = 0.847–0.959, *P* < 0.05) had lower risk of having high-quality embryos (Table 3). The logistic regression model was established and the formula was followed (in the formula, *p* indicates the probability of having high-quality embryo; x1 and x2 are explanatory variables, representing the values of age and Hcy, respectively).


(1) $$\begin{array}{r} p=\frac{1}{1+\exp\left[-\left(2.641-0.041x_{1}-0.104x_{2}\right)\right]} \end{array}$$

**Figure 3:**
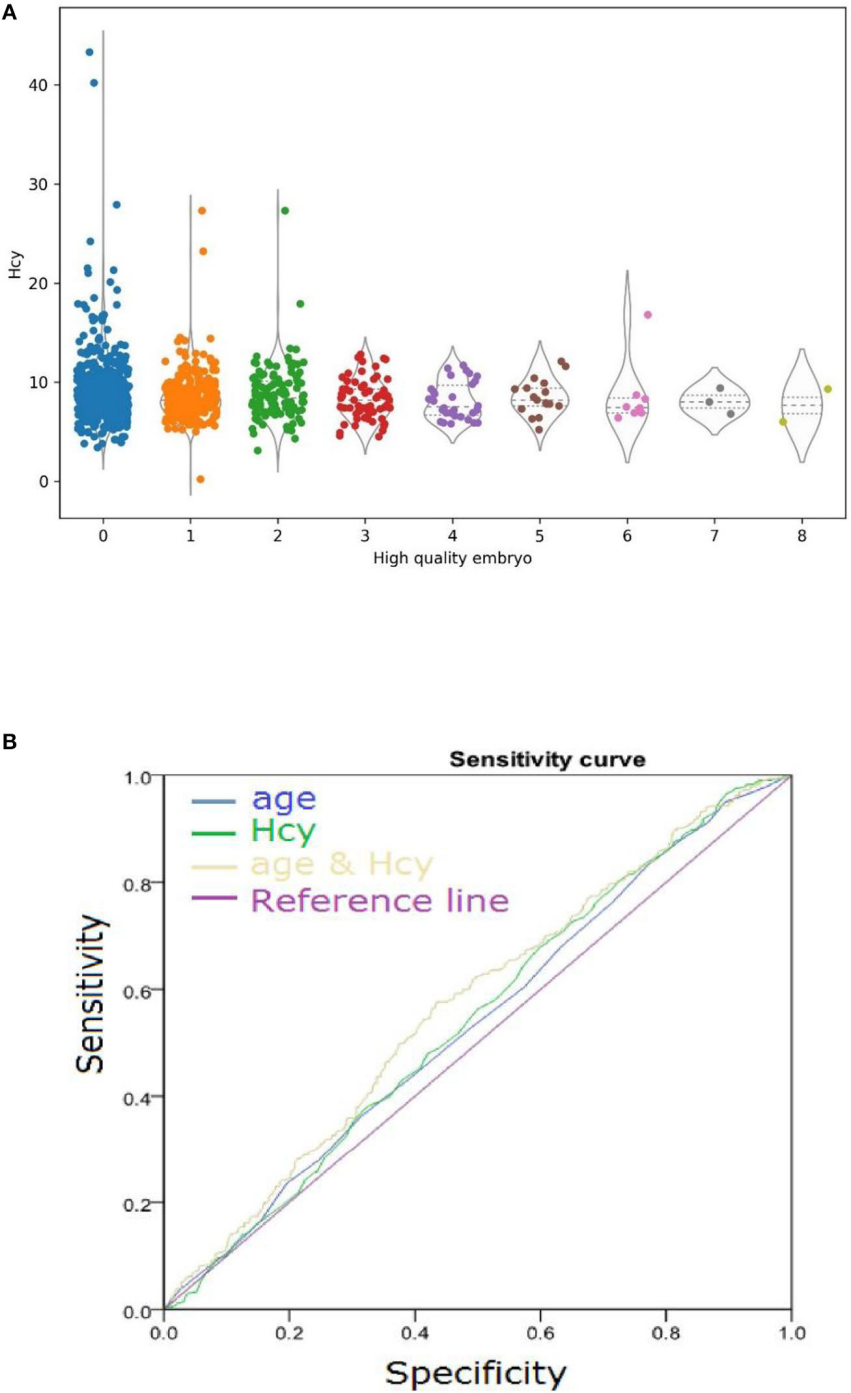
**(A)** Serum Hcy levels negatively correlated with high-quality embryo number. Horizontal lines represent the median and interquartile range (IQR) of the data. **(B)** Diagnostic Performance of embryo quality. Comparison of model performance for predicting embryo quality using variables age and Hcy independently and using variables age and Hcy simultaneously.

The AUC for using this formula to differentiate age and Hcy was 0.53 and 0.543, respectively (Figure 3).

**Table 3:** The results of logistic regression analysis.

**Variable**	**β**	**SE**	***P***	**OR (95% CI)**
Age	−0.041	0.019	0.028	0.960 (0.926 to 0.996)
Hcy	−0.104	0.032	0.001	0.901 (0.847 to 0.959)
β_0_	2.641	0.952	0.001	14.028

*n = 975. Missing data have not been imputed. In the variable screening process, the “backward: LR” method is used*.

## Discussion

According to the latest national census of China, the fertility rate significantly decreased in 2020 (1.3 children per woman). One reason is that more Chinese young people tend to postpone marriage and childbearing to later reproductive years; the other reason is that the number of infertile couples has increased (13, 14). Furthermore, the Chinese government released a two-child policy encouraging families to have a second child in January 2016. To sum up the above reasons, the reproductive need in women of advanced age (≥ 40) has been increasing dramatically, thus leading to higher demands for receiving ART in recent years. Unfortunately, DOR has become a huge challenge for reproductive medicine. The published results showed that about 20% of infertile women suffered from DOR in China (15–17). DOR induces not only infertility but also poor fertility outcomes even when *in vitro* fertilization-embryo transfer (IVF-ET) is used (18).

The goal of ART is to deliver a healthy baby. Therefore, it is essential to evaluate ovarian reserve, oocyte quality, and embryo quality. In clinical practice, there is a standard scheme for definite DOR, in which age, baseline AFC (<5–7), baseline AMH (<0.5~1.1 ng/ml), and baseline FSH (>10 mIU/ml) are considered (19, 20). However, the existing research showed that these parameters are not good predictors of embryo quality or pregnancy in controlled ovarian stimulation cycles in ART processing (6, 21, 22). Therefore, we designed a project to explore the potential correlation between embryo quality and potential parameters from medical records.

Our study showed that the level of serum AMH was significantly higher while PRL and Hcy were statistically lower in patients with high-quality embryos compared to those with poor-quality embryos. The role of AMH level as a biomarker of embryo quality remains controversial (23–26). Most researchers argued that serum AMH concentrations fluctuate for three days after stimulating with standard hormone-based drugs. PRL, a 198 amino acid polypeptide hormone, is secreted from the anterior pituitary. Its secretion could be affected by stress, pain, trauma, ingesting food, and pregnancy (27, 28). Hence, some researchers do not believe it might affect the outcome of IVF (29). Hcy, a sulfur-containing non-essential amino acid generated from the metabolism of the methionine and cysteine, plays a vital role in remethylation and trans-sulphuration. Previous studies have shown that a high serum Hcy level might lead to a reduction in the number of oocytes, suppress oocyte maturity, and reduce embryo quality (30–32). In order to reveal the correlation between AMH, PRL, Hcy, and embryo quality, a logistic regression analysis model was built for the analysis. The results demonstrated that only serum Hcy level correlated with embryo quality. In particular, a high Hcy level coupled with aging might partly reduce embryo quality. Our findings suggested that Hcy might be a sensitive marker for predicting embryo quality in patients with DOR of advanced age who underwent IVF. In other words, a high serum level of Hcy might reduce the embryo quality.

The Hcy is an intermediate product of methionine metabolism *via* methylation catalyzed by various enzymes, including betaine-homocysteine *S*-methyltransferase (BHMT), glycine *N-*methyltransferase (GNMT), guanidinoacetate *N-*methyltransferase (GAMT), cystathionine β*-*synthase (CBS), and γ*-*cystathionase (33, 34). These reactions depend on folate and B-vitamins, including vitamin B-2, B-6, and B-12 as cofactors at different steps of the metabolic pathways (35, 36). The main catabolic product of Hcy is cysteine, which is helped by CBS and γ*-*cystathionase. Thus, we can assume that a moderate supply of B-vitamin, folate, and cysteine in our diet might be beneficial to obtaining high-quality embryos in IVF (37). A high-methionine diet could induce hyperhomocysteinemia, which is related to some pathological statuses, including infertility (28).

In conclusion, the presented study demonstrated that high serum Hcy level is linked to poor-quality embryos. Moreover, Hcy coupled with age might be a sensitive marker for embryo quality after IVF. In connection with this result, we assumed that improving the metabolism of Hcy will benefit the success of assisted reproduction.

## Data Availability Statement

The original contributions presented in the study are included in the article/supplementary materials, further inquiries can be directed to the corresponding authors.

## Ethics Statement

The studies involving human participants were reviewed and approved by Dalian Municipal Women and Children's Medical Center (#No. 2020010). The Ethics Committee waived the requirement of written informed consent for participation.

## Author Contributions

Conceived and designed the experiments: HW, AH, XS, and JL. Performed the experiments: DC, YJ, LS, NZ, and ST. Analyzed the data: AH and SJ. Contributed reagents, materials, and analysis tools: XS, JL, XX, and ST. Wrote the manuscript: HW, AH, and JL. The final manuscript was read and approved by all authors.

## Conflict of Interest

The authors declare that the research was conducted in the absence of any commercial or financial relationships that could be construed as a potential conflict of interest.

## Publisher's Note

All claims expressed in this article are solely those of the authors and do not necessarily represent those of their affiliated organizations, or those of the publisher, the editors and the reviewers. Any product that may be evaluated in this article, or claim that may be made by its manufacturer, is not guaranteed or endorsed by the publisher.

## Abbreviations

Hcy: homocysteine
DOR: Diminished ovarian reserve
IVF: *in vitro* fertilization
ICSI: Intracytoplasmic sperm injection
AMH: Anti-Müllerian hormone
FSH: Follicle stimulating hormone
AFC: Antral follicle count
POR: Poor ovarian response
POF: Premature ovarian failure
POI: Primary ovarian insufficiency
LH: Luteinizing hormone
PRL: Prolactin
E_2_: Estradiol
CHO: Cholesterol
TG: Triglyceride
LP: Lipoprotein
LDL: Low density lipoprotein
ART: Assisted reproductive technique
BMI: Body mass index
Gn: Gonadotrophin
2PN: Two pronuclear
IQR: interquartile range
hHcy: Hyper-homocysteinemia.
